# DPB-NBFnet: Using neural Bellman-Ford networks to predict DNA-protein binding

**DOI:** 10.3389/fphar.2022.1018294

**Published:** 2022-10-28

**Authors:** Jing Li, Linlin Zhuo, Xinze Lian, Shiyao Pan, Lei Xu

**Affiliations:** ^1^ School of Data Science and Artificial Intelligence, Wenzhou University of Techonology, Wenzhou, China; ^2^ School of Electronic and Communication Engineering, Shenzhen Polytechnic, Shenzhen, China

**Keywords:** bioinformatics, DNA-protein binding, graph neural networks, neural Bellman-Ford algorithms, DPB-NBFnet, computational biology

## Abstract

DNA is a hereditary material that plays an essential role in micro-organisms and almost all other organisms. Meanwhile, proteins are a vital composition and principal undertaker of microbe movement. Therefore, studying the bindings between DNA and proteins is of high significance from the micro-biological point of view. In addition, the binding affinity prediction is beneficial for the study of drug design. However, existing experimental methods to identifying DNA-protein bindings are extremely expensive and time consuming. To solve this problem, many deep learning methods (including graph neural networks) have been developed to predict DNA-protein interactions. Our work possesses the same motivation and we put the latest Neural Bellman-Ford neural networks (NBFnets) into use to build pair representations of DNA and protein to predict the existence of DNA-protein binding (DPB). NBFnet is a graph neural network model that uses the Bellman-Ford algorithms to get pair representations and has been proven to have a state-of-the-art performance when used to solve the link prediction problem. After building the pair representations, we designed a feed-forward neural network structure and got a 2-D vector output as a predicted value of positive or negative samples. We conducted our experiments on 100 datasets from ENCODE datasets. Our experiments indicate that the performance of DPB-NBFnet is competitive when compared with the baseline models. We have also executed parameter tuning with different architectures to explore the structure of our framework.

## 1 Introduction

DNA is a hereditary material that plays an essential role in human metabolism and almost all organisms. Meanwhile, proteins are a vital composition and principal undertaker of microbe movement. Therefore, studying the interactions between DNA and proteins is highly significant from the biological point of view because the influence of DNA-binding proteins on a large number of biological processes is conclusive, especially in gene transcription and regulation. However, traditional experimental methods to detect DNA-protein binding (DPB), such as CHIP-seq or new methods such as ProNA 2020 (Qiu et al., 2020), are extremely expensive and time consuming. To cut the cost, computational biologists have used the deep learning architecture to predict the binary label of sequence-based data, which indicates the relationship of sequences. These learning tasks often have large amount of training examples, which allow scientists to adapt them to deep learning structures, especially graph neural networks (GNNs), without experiencing the over-fitting problem.

Many deep learning architectures have been used to predict DNA-protein binding (Dong et al., 2022). For example, DeepRAM (Trabelsi et al., 2019) first obtained the sequence representation by word2vec embedding, and then used convolutional layers and recurrent layers to process the data. DeepBind built a Long-Short-Term-Memory (LTSM) and Convolutional Neural Network (CNN) structure to model the sequence data (Alipanahi et al., 2015). Hierarchical Attention Networks (HANs) are another kind of architecture that is based on the natural language processing method for document classification (Yu et al., 2019). Trabelsi et al. (2019) demonstrated a comprehensive evaluation of deep learning architectures, including CNN (Zeiler and Fergus, 2014) and Recurrent Neural Networks (RNNs; Medsker and Jain, 2001), to predict DNA/RNA binding specificities.

Computational biologists have recently built GNNs to predict DNA- or RNA-protein interactions. For example, Guo et al. (2021) developed a method called DNA-GCN, which utilized a graph CNN architecture to first build a large graph containing the neighborhood information and then turn this problem into a node classification task. In another example, Shen et al. (2021) developed a method called NPI-GNN (which is composed of GraphSAGE, top-k pooling, and global pooling layers) to predict non-coding RNA (ncRNA)-protein interactions. Other work related with predicting molecular interactions has also built a knowledge graph and then utilized a GNN model (Song et al., 2022; Zeng et al., 2022).

Despite these studies, there is still a gap in the research of predicting DNA-protein binding with GNNs. In general, predicting DNA-protein binding could be regarded as a link prediction problem on a graph. In this graph, we regard different DNA and proteins as vertices with different attributes. After building the graph, we transfer predicting DNA and protein binding into predicting the edge existence of different vertices, which is a link prediction problem in a homogeneous graph.

We now wonder if we could combine the advanced methodology for link prediction problem with our real biological data. In our work, we propose a novel GNN method, which is called DPB-NBFnet and is based on Neural Bellman-Ford neural networks (NBFnets) (Zhu et al., 2021). The NBFnet is a novel GNN architecture that was developed by Zhu for the link prediction problem, which unified the link prediction methods in both heterogeneous and homogeneous graphs. NBFnet includes three neural functions—IND (INDICATOR), MES (MESSAGE), and AGG (AGGREGATE) functions—and it has been proved to have a state-of-the-art performance when compared to other methods for the link prediction problem, including GraIL (Teru et al., 2020). We evaluated our model on the 100 chosen datasets from ENCODE. Compared with other GNN models for the link prediction problem, the final accuracy and time consumption of our DNA-NBFnet framework for prediction of DNA-protein binding have been shown to be superior. We believe that our work could make some contributions to the study of DNA-protein binding and will also inspire other computational biological models.

## 2 Related work

### 2.1 Homogeneous and heterogeneous graphs

Given the real sequence data of DNA and protein, we first obtain a graph in which each vertex represents a DNA or a protein, and each edge represents an interaction or binding between DNAs and proteins. In this way, we could keep the topological structure of initial data (Cai et al., 2020b). Generally, a graph (West et al., 2001) is denoted as an ordered triplet (V,E,R) . This triplet is composed of a nonempty set V representing vertices, a set E representing edges, and R representing the relation types. Moreover, we use N(u) to denote the set of neighborhood nodes of node *u*, E(u) to denote the set of edges whose endpoint is *u*. If there are various kinds of nodes or edges, then this graph is categorized as a heterogeneous graph, which is represented as (V,E,R) , otherwise it is categorized a homogeneous graph (V,E) . In our study, we build a homogeneous graph where we initialize DNA and protein as the same kind of vertices but with different labels in actual node representations. After building our graph, we turn predicting the DNA-protein binding into a link prediction problem between these nodes. Our goal is to predict the edge existence between different vertices of this graph.

### 2.2 Different methods for link prediction problems

There are currently three methods to solve this link prediction problem in our graph, which are path-based methods, embedding methods, and GNNs (Liu et al., 2022a; Liu et al., 2022b; Peng et al., 2022). Among these methods, GNNs are a growing family of methods and have shown the most advanced performance. GNN models are a set of representation learning functions. The highlight of GNNs is that they have the ability to encode topological structures of graphs (Cai et al., 2020a; Wu et al., 2020a; Fu et al., 2020). There are several kinds of GNNs for DNA-protein link prediction problem. DNA Graph Convolution Networks (DNA-GCN) encapsulate each node’s hidden representation by aggregating the feature information from its neighbors (Guo et al., 2021). DNA Graph Attention Networks (DNA-GANs) adopt attention mechanism to aggregating feature information and concatenate the outputs of multiple models (Xiao et al., 2017). However, these methods require the information of global graph and can only be used in transductive learning. Variational Graph Auto-encoders (VGAE) learns a graph embedding to get node embeddings for all nodes, and then aggregates the embeddings of the source and target nodes as their link representation (Kipf and Welling, 2016). These frameworks encode node representations by different GNN models and decode edges as functions over node pairs. SEAL is another mainstream framework, which has an end-to-end structure that encodes the enclosing subgraphs of each node explicitly. However, these structures require a subgraph to be created for each node, resulting in a high cost for large graphs, especially in DNA-protein binding graphs. In comparison with these methods, our DNA-NBFnet encapsulates the paths between two vertices at a relatively low cost.

### 2.3 Path formulation for DNA-protein binding prediction

The goal of link prediction is to predict the existence of a relationship *r* between two vertices *u* and *v*. For DNA-protein binding, the vertices could be proteins or DNA and the relationship is their binding. To capture the paths between two nodes, we regard it as a message passing process, and its power for link prediction has been proven by previous work. For example, PageCon considers different edge features without node difference in the graph and then passes relational messages among edges iteratively to aggregate neighborhood information (Wang et al., 2021). For a given entity pair *u* and *v*, PageCon models neighborhood topology including relational context and relational paths, and combines them for link prediction. In another example, MPNN learns representations on graph data by a message passing algorithm and executes an aggregation procedure to compute a function of their entire input graph (Gilmer et al., 2020). Based on these inspirations, our method combines the message passing heuristics and Bellman-Ford algorithm to efficiently solve the link prediction problem.

In our case, we only consider one kind of relation (i.e., if a DNA-protein binding exists). This requires a pair representation **h** (*u*, *v*) to be learned. Inspired by the message passing methods, this algorithm should pass relational messages among edges iteratively and finally aggregate neighborhood information. This pair representation should capture the local topological structure between the nodes *u* and *v*. Traditional methods, such as the Katz index (Katz, 1953) and PageRank (Page et al., 1999), encode such structure by counting various kinds of random walks from *u* to *v*. Based on this intuition, the pair representation **h** (*u*, *v*) is formulated as a generalized sum of path representations between these two nodes, with ⊕ being a summation calculator. Each path representation is formulated as a generalized product of edge representations in the path between them with the multiplication operator ⊗.

$$h\left(u,v\right)=h\left(P_{1}\right)⊕h\left(P_{2}\right)⊕\cdots ⊕h\left(P_{\left|P_{uv}\right|}\right)≔\overset{P_{uv}}{\underset{i=1}{⊕}}h\left(P_{i}\right)$$ (1)

$$h\left(P_{i}=\left(e_{1},e_{2},\ldots ,e_{\left|P_{i}\right|}\right)\right)=w\left(e_{1}\right)⊗w\left(e_{2}\right)⊗\cdots ⊗w\left(e_{\left|P_{i}\right|}\right)≔\overset{\left|P_{i}\right|}{\underset{j=1}{⊗}}w\left(e_{j}\right)$$ (2)

where $P_{uv}$ represents the set of paths from *u* to *v*, $h\left(P_{i}\right),\left(i=1,2,\ldots ,P_{|P_{uv}}|\right)$ represents the numbered path representation from *u* to *v*, and each path *P*_*i*_ is composed of several edges $e_{1},e_{2},\ldots ,e_{\left|P_{i}\right|}$ **w** (*e*_*j*_) denotes the representation of *j*-th edge *e*_*j*_ on the path **h** (*P*_*i*_). In our formulation, there are two operators, a multiplication operator ⊗ and a summation operator ⊕. We need ⊕ to be commutative, but ⊗ not because it is defined to compute the exact order of the product.

The path formulation could be interpreted explicitly as follows. We first search all possible paths from *u* to *v*. We then compute the path representations by multiplication (Equation 2). Finally, we aggregate the path representations as the final pair representation (Equation 1). This path formulation is able to model several traditional link prediction methods, including PageRank and Katz index, which has been proven (Zhu et al., 2021).

### 2.4 Generalized Bellman-Ford algorithm

As shown in Equations 1, 2, the number of paths increases exponentially as the length of the path grows. Therefore, the computational cost grows drastically. Here, a flexible solution is provided using the generalized Bellman-Ford algorithm. Assuming that the multiplication operator ⊗ and summation operator ⊗ satisfy the semi-ring system (Rowen, 2012), with multiplication identity *{1}○* and summation identity *{0}○* respectively, we have the following algorithm, which is called the generalized Bellman-Ford algorithm.

$$h^{\left(0\right)}\left(u,v\right)\leftarrow 1_{\left(u=v\right)}$$ (3)

$$h^{\left(t\right)}\left(u,v\right)\leftarrow \left(\underset{\left(x,v\right)\in \varepsilon \left(v\right)}{⊕}h^{\left(t-1\right)}\left(u,x\right)⊗w\left(x,v\right)\right)⊕h^{\left(0\right)}\left(u,v\right)$$ (4)

where **1**_(*u*=*v*)_ is an indicator function. We define it to be equal to *{1}○* if *u* = *v* and *{0}○* in other cases. **w** (*x*, *v*) is the representation for edge *e* = (*x*, *v*). Equation 3 is also called the boundary condition and Equation 4 is called the Bellman-Ford iteration. It has been proven that this algorithm can solve the traditional algorithm, including graph distance, Katz index, widest path and most reliable path algorithms, personalized PageRank with different multiplication, and summation operators (Zhu et al., 2021).

In summary, this algorithm is able to obtain a pair representation **h** (*u*, *v*) for a given node *u* and all v∈V . This method reduces the computational costs by the distributive property of multiplication over summation. Because *u* and *r* are fixed, we can abbreviate **h** ^(*t*)^(*u*, *v*) as $h_{v}^{\left(t\right)}$ . Finally, we get a source-specific pair representation $h_{v}^{\left(t\right)}$ .

## 3 Methodology

### 3.1 Data representations

We collected 100 datasets from ENCODE datasets. ENCODE is an encyclopedia of DNA datasets, which contains about 503,038 datasets in total. From an economical and practical view, we randomly chose 100 datasets among them. Each dataset is related to one specific DNA-binding protein like regulation factor or transcription factor. For each protein, amino acids are divided into seven groups. The datasets contain both positive and negative samples, among which the positive ones were DNA sequences that were experimentally verified and confirmed to bind to this protein. The negative samples were generated by corrupting one of the entities of these positive samples. After collecting the datasets, we get the graph data representation using large matrix *M*, where *M* contains the vertex information, topological information and true label *l*_*i*_ (being positive or negative) for the *i*-th sample.

### 3.2 Neural parametrization

Given the source node *u* and the number of setup layers *T*, the neural Bellman-Ford networks output the pair representation **h** (*u*, *v*). We parameterize the generalized Bellman-Ford algorithm with three neural functions—which are called IND function, MES function and AGG function here—and we get the following NBFnet algorithm:


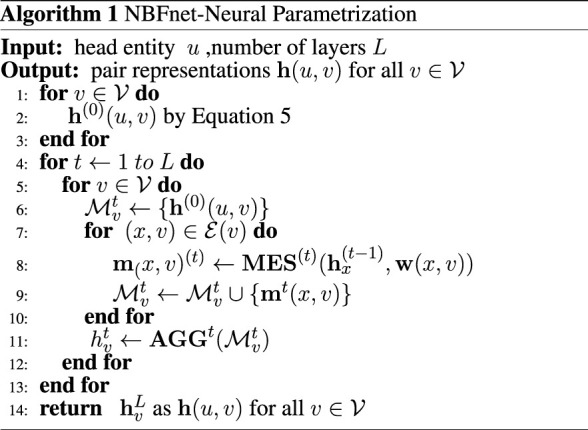


By substituting the neural functions **IND**, **MES**, **AGG** for normal functions in Equations 3, 4, we get the final formulas of NBFnet:

$$h^{\left(0\right)}\left(u,v\right)\leftarrow IND\left(u,v\right)$$ (5)

$$h^{\left(t\right)}\left(u,v\right)\leftarrow AGG\left(\left\{MES\left(h_{x}^{\left(t-1\right)},w\left(x,v\right)\right)|\left(x,v\right)\in E\left(v\right)\right\}⋃\left\{\overset{\left(0\right)}{\underset{v}{h}}\right\}\right)$$ (6)

In general, NBFnet could be regarded as a kind of GNN structure for learning pair representations. These neural functions (i.e., IND, MES, AGG functions) remain to be learned.

### 3.3 SortPooling

After obtaining the pair representations **h** (*u*, *v*) (given the head entity *u*) *via* our NBFnet, we process the data with a feed-forward neural network *f* (⋅). This network is first is built by a leaky rectified linear unit (ReLU) layer and then a SortPooling layer. The SortPooling layer, unlike simple global pooling layers (Zhang M. et al., 2018), is able to cut down the size of the graph in a flexible and smart manner and effectively extract features. In this pooling layer, the input is sorted by WL colors and it imposes the output as consistent ordering graph vertices. Assuming that the input of these layers $\tilde{h}\left(u,v\right)$ is an *N* × *d* dimensional matrix, the output of the SortPooling layer is a *K* × *d* dimensional matrix. Here, *K* is a self-defined integer. SortPooling layer output the most refined *k* vertices by WL colors (Shervashidze et al., 2011; Wu et al., 2021b,a).

### 3.4 AlphaMEX: Global pooling

Unlike using a normal global pooling layer using the average function or maximum function to replace the whole layer, AlphaMEX is an end-to-end global pooling operator where a nonlinear log-mean-exp function is set up to more effectively process features (Zhang B. et al., 2018; Wu et al., 2020b; Qi et al., 2022). In DPB-NBFnet, we use the AlphaMEX to cut down the size of the graph because the whole network structure contains more layers. The AlphaMEX introduces a parameter *α*, whose function formula is designed as follows:

$${AlphaMEX}_{\alpha }\left\{h_{i}\right\}≔\frac{1}{\log\left(\frac{\alpha }{1-\alpha }\right)}\log\left(\frac{1}{n}\overset{n}{\underset{i=1}{\sum }}{\left(\frac{\alpha }{1-\alpha }\right)}^{h_{i}}\right)$$ (7)

where **h**_*i*_ denotes input matrix and *n* denotes the numbers of **h**_*i*_. *α* is limited from 0 to 1, which is a trainable parameter.

Together with NBFnet, the leaky ReLU and Sortpooling layer are consecutively composed of one feature extracting module. In our framework, we set up three feature extracting modules sharing this structure. We then process the data with three global pooling layers, here we implement AlphaMEX, which is a better global pooling operator for convolutional neural networks. The additive module then sums up the results of the AlphaMEX pooling layers. Finally, the output of the additive module is presented to a network of fully connected layers. The outcome of the last FC layer is processed by a softmax function. We then take the logarithmic value as the final predicted result.

**FIGURE 1:**
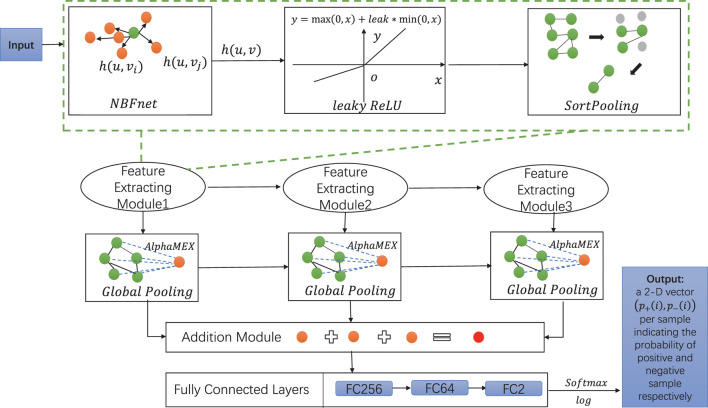
The framework of DPB-NBFnet is composed of three feature processing modules, each of which contains NBFnet, leaky ReLU (set leak as 0.2), and SortPooling. Consecutively, the framework contains three global pooling layers, one addition module, and FC layers (fully connected layers) with 256,64,2 neurons. A homogeneous graph is first input to the feature extracting module. The output is then processed by the following two feature extracting modules. The output of three feature extracting modules is then read by three AlphaMEX global pooling layers, whose outputs are put together by the addition module. The outcome of the addition module is then read by three FC layers with 256, 64, and 2 neurons, respectively. We then use a softmax function then a logarithmic function to give the final predicted value *p*_+_(*i*) and *p*_−_(*i*) to classify the positive and negative samples, respectively.

Eventually, we get a 2-D vector (*p*_+_(*i*), *p*_−_(*i*)) that indicates the probability of the positive sample and negative sample, respectively. The overall workflow of DPB-NBFnet framework is illustrated in Figure 1.

### 3.5 Loss function

For the i-th DNA-protein pair, if there is a binding in this pair, then we denote it as a positive sample, and otherwise negative. We aim to minimize the negative logarithmic likelihood of positive and negative pairs. Hence, we design and use such a loss function in our model:

$$L=-\frac{1}{k}\overset{k}{\underset{i=1}{\sum }}\left[l_{i}p_{+}\left(i\right)+\left(1-l_{i}\right)p_{-}\left(i\right)\right]$$ (8)

where *k* is the number of samples in the dataset, and *l*_*i*_ the binary label of the *i*-th sample.

### 3.6 Time complexity

DPB-NBFnet has a relative low time complexity compared with other GNN frameworks. We will discuss its time complexity roughly. Assuming that our model needs to infer the likelihood of a dataset containing |V| samples with *d* dimensions, where V is the set of all uncertain positive and negative samples, we need to implement the algorithm (Equations 5, 6) once to get the predictions. The time complexity here is O(|E|d) . After this, a constant *K* is settled for Equations 5, 6 to converge. So far, it has a time complexity $O\left(|E|d+|V|d^{2}\right)$ . In summary, for each sample, the average time complexity is $O\left(\frac{|E|d}{V}+|d^{2}\right)$ .

### 3.7 Measurements and evaluation

In our work, we utilize the average precision (AP), the average recall (AR), the average accuracy (AC), the Matthew’s correlation coefficient (MCC; Chicco and Jurman, 2020), and the area under the receiver operating characteristic curve (AUROC; Hosmer et al., 2013) to measure the performance of our DPB-NBFnet modules. These statistics are defined as follows:

$$PR=\frac{TrP}{TrP+FaP}$$ (9)

$$RE=\frac{TrP}{TrP+FaN}$$ (10)

$$AC=\frac{TrP+TrN}{TrP+TrN+FaP+FaN}$$ (11)

$$MCC=\frac{TrP\cdot TrNFaP\cdot FaN}{\sqrt{\left(TrP+FaP\right)\left(TrP+FaN\right)\left(FaN+FaP\right)\left(TrN+FaN\right)}}$$ (12)

Where *TrP*, *TrN*, *FaP*, *FaN* denotes the number of true positive samples, true negative samples, false positive samples, and false negative samples, respectively. The AUROC is calculated by its geometric meaning by Python, namely the area under the ROC curve (Wu et al., 2021c; Su et al., 2022).

## 4 Experiment

### 4.1 Experiment setup

We collected 100 datasets from ENCODE datasets. Each of these datasets corresponds to a specific DNA-binding protein like transcription factor or regulation factor. The positive samples are DNA sequences that were experimentally confirmed to bind to this protein, and the negative samples were generated by corrupting one of the entities of these positive samples.

After preparing the data, we implement the DPB-NBFnet by Python, with the main packages PyTorch 1.10.0 and PyTorch-Geometric (PyG) 2.0.4. PyG is a library for implementing GNN models on structured data. Given that our NBFnet can be regarded as a GNN model, it is convenient for us to use this package.

### 4.2 Main results

We evaluated the DNA-protein binding prediction performance of our DPB-NBFnet framework on the 100 ENCODE datasets. We used a four-fold cross-validation strategy. We compared DPB-NBFnet model with other link prediction models, among which we chose a traditional path-based method Katz index, an embedding method node2vec (Grover and Leskovec, 2016), and three GNN models: VGAE (Kipf and Welling, 2016), DRUM (Sadeghian et al., 2019), and SEAL (Zhang and Chen, 2018). Table 1 presents the final prediction results. As can be seen in Table 1, our DPB-NBFnet framework was able to achieve 93.7% precision, 92.6% recall, 95.2% accuracy. Therefore, it has shown a state-of-the-art performance compared with other methods for link prediction problems on DNA-protein binding.

**TABLE 1:** Performance comparison of different methods on ENCODE datasets.

Methods	PR^a^ (%)	RE^b^ (%)	AC^c^ (%)	MCC^d^	AUROC^e^ (%)
Katz index	81.2	72.5	81.0	74.9%	75.7
node2vec	83.4	76.6	87.2	89.6%	89.1
VGAE	91.0	90.0	92.0	86.8%	94.7
DRUM	91.7	90.5	93.7	83.9%s	96.9
SEAL	91.5	91.3	91.4	82.71%	97.5
DPB-NBFnet	93.7	92.6	97.2	89.1%	98.2

^a^ PR stands for precision (Equation 9).

^b^ RE stands for recall (Equation 10).

^c^ AC stands for accuracy (Equation 11).

^d^ MCC stands for Matthew correlation coefficient (Equation 12).

^e^ AUROC stands for area under the receiver operating characteristic curve (Hosmer et al., 2013).

### 4.3 Exploration of the DPB-NBFnet structure

#### 4.3.1 Neural functions

In general, DPB-NBFnet benefits from advanced embedding methods, such as DistMult (Yang et al., 2017), RotatE (Sun et al., 2019) and TransE (Bordes et al., 2013). Compared with explicit AGG functions (i.e., sum, max, mean), combinations of advanced AGG and MES functions achieve a better performance. Table 2 gives the results of AUROC choosing different MES and AGG functions.

**TABLE 2:** AUROC results of different AGG and MES functions.

AGG\MES	Sum (%)	Mean (%)	Max (%)
DistMult	85.8	89.3	90.7
TransE	89.2	74.7	93.6
RotatE	94.6	96.5	98.4

#### 4.3.2 Number of GNN layers

As can be seen in Algorithm 1, parameter *L* is required as an input, which represents the number of layers. Although some studies have reported that the GNN model usually has a better performance when the layers go deeper, we observed that the DPB-NBFnet does not behave in this way. At first the performance improves as more layers are included but it then reaches saturation at about six layers. We predict that this happen because paths no longer than six are enough for a link prediction problem. The results of AUROC are listed in Table 3.

**TABLE 3:** Results of different AGG and MES functions with multiple measurements.

#layers T\Methods	2 (%)	3 (%)	4 (%)	5 (%)	6 (%)	7 (%)	8 (%)
PR^a^	71.2	77.2	80.1	81.5	85.8	96.1	96.3
RE^b^	73.4	79.5	81.4	85.6	90.4	96.8	97.0
AC^c^	75.8	80.4	83.4	85.8	92.1	97.2	97.4
MCC^d^	73.3	82.1	85.4	86.6	91.4	96.9	97.1
AUROC^e^	72.6	81.4	86.4	91.3	94.4	97.6	97.6

^a^ PR stands for precision (Equation 9).

^b^ RE stands for recall (Equation 10).

^c^ AC stands for accuracy (Equation 11).

^d^ MCC stands for Matthew correlation coefficient (Equation 12).

^e^ AUROC stands for area under the receiver operating characteristic curve (Hosmer et al., 2013).

## 5 Discussion and conclusion

### 5.1 Application

Predicting DNA-protein binding has a significant meaning from the micro-biological point of view, but it is extremely expensive to explore all kinds of DNA-binding proteins *via* experimental methods. Our DPB-NBFnet framework was able to achieve a prediction accuracy of 97.2%, which could be applied into predictions of DNA-protein binding on real datasets. This provides biologists with a cost-effective method to explore more DNA-binding proteins and also study their actual functions in micro-organisms.

### 5.2 Conclusion

In this work, we present a novel framework DPB-NBFnet, which is a GNN model that predicts DNA-protein binding. This framework uses NBFnet, SortPooling, and AlphaMEX, which are all technologies from modern machine-learning research. Our results show that DPB-NBFnet outperformed baseline models. We also explore the influence of different neural functions and number of layers on our DPB-NBFnet structure. DPB-NBFnet can be considered to be a substitute option to the existing link prediction methods on real datasets. We also hope that this method could inspire more computational biologists and could be put into use in diverse kinds of tasks in the future.

## Data Availability

The original contributions presented in the study are included in the article/Supplementary Material, and further inquiries can be directed to the corresponding authors.
